# The architectural characteristics of the hamstring muscles do not differ between male and female elite-level rugby union players

**DOI:** 10.3389/fphys.2023.1129061

**Published:** 2023-01-27

**Authors:** Kevin Cronin, Shane Foley, Seán Cournane, Giuseppe De Vito, Fearghal Kerin, Garreth Farrell, Eamonn Delahunt

**Affiliations:** ^1^ School of Medicine, University College Dublin, Dublin, Ireland; ^2^ School of Physics, University College Dublin, Dublin, Ireland; ^3^ Department of Biomedical Sciences, University of Padova, Padua, Italy; ^4^ Leinster Rugby, Dublin, Ireland; ^5^ School of Public Health, Physiotherapy and Sports Science, University College Dublin, Dublin, Ireland; ^6^ Institute for Sport and Health, University College Dublin, Dublin, Ireland

**Keywords:** ultrasound, hamstring architecture, injury, female athlete, male athlete

## Abstract

**Purpose:** To determine whether differences exist in the architectural characteristics of the hamstring muscles of elite-level male and female rugby union players.

**Methods:** Forty elite-level rugby union players (male n = 20, female n = 20) participated in this cross-sectional study. A sonographer acquired static ultrasound images using a 92 mm linear transducer to quantify (*via* a semi-automated tracing software tool) the architectural characteristics (muscle length, fascicle length, pennation angle, and muscle thickness) of the biceps femoris long head and semimembranosus muscles of participants’ left limb. Muscle length and muscle thickness of the biceps femoris short head and semitendinosus muscles of participants’ left limb were also quantified. Bonferroni adjusted independent samples t-tests were performed to evaluate whether differences exist in the architectural characteristics of the hamstring muscles of elite-level male and female rugby union players.

**Results:** There were no significant differences in fascicle length or pennation angle of the hamstring muscles of elite-level male and female rugby union players. Some significant differences in muscle thickness (biceps femoris short head, and semimembranosus) and muscle length (biceps femoris short head, biceps femoris long head, semitendinosus, and semimembranosus) were observed; in all cases the male players had thicker and longer muscles.

**Conclusion:** At a group level, hamstring muscle fascicle length and pennation angle are unlikely to be a sex-specific intrinsic risk factor for Hamstring strain injuries.

## Introduction

In professional rugby union, a negative association between injuries and team success has been reported (Williams et al., 2017). Therefore, in order to enhance the assets of the sport organisation, a primary focus needs to be placed on injury prevention. Hamstring strain injuries (HSI) have consistently been identified as one of the most common injuries sustained by elite-level rugby union players (Fuller et al., 2013; Fuller et al., 2017). Indeed HSI were reported to account for 9.8% of all injuries incurred during the (men’s) 2019 Rugby World Cup (Fuller et al., 2020). A similar injury prevalence has been reported in other rugby union injury surveillance studies (Fuller et al., 2013; Fuller et al., 2017), where HSI were the most common injury type sustained during matches. Both the injury burden and recurrence rate of HSI are high (Kerin et al., 2022).

It is generally accepted that male athletes are more likely to sustain acute HSI than their female counterparts (Blackburn et al., 2008; Blackburn and Pamukoff, 2013; Cross et al., 2013). Studies that directly compare the rate of injuries between male and female athletes have reported that male athletes are two to four times more likely to incur HSI than their female counterparts (Cross et al., 2013; Dalton et al., 2015; Edouard et al., 2016; Larruskain et al., 2018). In soccer, incidence rates for HSI vary between sexes; the incidence rate in men’s professional football is higher (1.5/1,000 h) than the incidence rate in women’s professional football (0.2/1,000 h) (Larruskain et al., 2018). However, HSI have been reported to be a significant concern for female athletes. A study conducted in women’s Gaelic football reported that HSI are common, and account for up to 22% of all injuries, with an associated injury burden of 66 days lost per 1,000 h (O'Connor et al., 2021). Other studies have reported that HSI are the second most common injury sustained by professional female soccer players (Crossley et al., 2020), and the third most frequent injury sustained by amateur female soccer players (Söderman et al., 2001). A similar injury prevalence (6%) has been reported in a women’s rugby union injury surveillance study (Williams et al., 2019). As detailed in the report of Williams et al. (2019), HSI resulted in a substantial number of days lost to injury (mean range 91–100 days).

Although HSI are a prevalent injury incurred by male and female athletes, little is known about potential inherent sex differences in the architectural characteristics of the hamstring muscles. Muscle architecture refers to the arrangement of fascicles within a given muscle, which ultimately governs the mechanical function of the muscle (Lieber and Ward, 2011). Aberrancies in the architecture of the bicep femoris long head muscle have been investigated as a potential intrinsic risk factor for HSI (Timmins et al., 2015). During fast eccentric activity (i.e., sprinting), the muscle undergoes active lengthening (Blazevich and Sharp, 2006). During this type of contraction, long muscle fascicles, compared to short muscle fascicles, exhibit less strain per sarcomere in series (Blazevich and Sharp, 2006); this increases the maximum shortening velocity of the muscle and has been proposed to reduce the risk of injury (Timmins et al., 2016a). One prospective study has reported that “short” bicep femoris long head muscle fascicles and low levels of eccentric knee flexor strength are risk factors for HSI in elite soccer players (Timmins et al., 2016a).

Ultrasound can be used to accurately and reliably quantify the architectural characteristics of skeletal muscle (Franchi et al., 2020). One key limitation of standard B-mode ultrasound transducers is their relatively narrow field of view (4–6 cm). Previous studies (Timmins et al., 2016a; Bourne et al., 2017) have assessed hamstring muscle architecture with narrow fields of view. These fields of view are typically shorter than the fascicles that are being measured. In these cases, fascicle length has been estimated (>50%) with various linear approximations, using the measured muscle thickness and pennation angle values (Timmins et al., 2016a; Bourne et al., 2017). Thus, the ability to quantify the architectural characteristics of the hamstring muscles is dependent upon the accuracy and repeatability of the measurement technique (Cronin et al., 2022). Recently, a semi-automated tracing software tool was develop to quantify the architectural characteristics of the hamstring muscles (Cronin et al., 2021). This tracing software tool precisely measures fascicle length, whilst accounting for fascicle curvature (Cronin et al., 2021).

Sex differences in lower extremity kinanthropometry and anatomical structure have been reported to account for differences in rates of knee injuries sustained by male and female athletes (Mendiguchia et al., 2011). However their potential association with sex differences in the rate of HSI has yet to be explored (Tillman et al., 2005). One study conducted by Behan and others identified that the fascicle length of the biceps femoris long head muscle did not differ between males and females (Behan et al., 2019). However a comparison of the architectural characteristics of the hamstring muscles of elite-level male and females athletes has yet to be reported in the published literature. Therefore, the purpose of this study was to determine whether differences exist in the architectural characteristics of the hamstring muscles of elite-level male and female rugby union players. We hypothesised that males would have longer biceps femoris long head and semimembranosus fascicle lengths compared to their female counterparts.

## Materials and methods

### Participants

Forty professional rugby union players were recruited by convenience sampling to partake in this cross-sectional study; 20 males (age = 24.5 ± 3.3 years; height = 1.9 ± 0.1 m; body mass, 100.4 ± 12.1 kg) and 20 females (age = 24.4 ± 3.3 years; height = 1.7 ± 0.1 m; body mass, 74.5 ± 9 kg). Using G* Power (statistical power analysis tool) to calculate the sample size for a cross sectional study with the following parameters, effect size (d) = 0.50, α err prob = 0.05, power (β err prob) = 0.4, a sample size of 40 participants was calculated (Faul et al., 2007). All participants provided written informed consent before the ultrasound assessment. Ethical approval for the study was granted by the UCD Human Research Ethics Committee (LS-21-50-Kerin-Delahunt).

### Hamstring ultrasound acquisition and digitisation

All sonograms were acquired by a single operator using a Hitachi Noblus ultrasound scanner (Hitachi Medical Systems, United Kingdom) with a 92 mm wide field of view transducer (Hitachi EUP-L53L). Trapezoid imaging was implemented at the beginning of each ultrasound scan to induce an image base wider than the footprint of the transducer; this permitted the inclusion of more lateral muscle architecture when measuring fascicles in the longitudinal plane up to 100 mm for a depth of 80 mm (Cronin et al., 2022).

During the acquisition of the sonograms, participants were positioned prone lying on a physiotherapy plinth. Participants’ legs were adjusted so that the medial aspect of the thigh was aligned with a previously placed mark on the plinth. A small amount of coupling gel was placed on the posterior thigh to allow transmission of the ultrasound waves intramuscularly. Ultrasound images were analysed using a semi-automated tracing software tool to quantify the architectural characteristics of the hamstring muscles (Cronin et al., 2021). Fascicle length was measured in centimetres where a fascicle was clearly sonographically illustrated extending from the intermediate aponeurosis to the superficial aponeurosis in the longitudinal plane (Cronin et al., 2021). The angle formed between the chosen fascicle and the intermediate aponeurosis is considered the pennation angle (Cronin et al., 2021). Muscle thickness was defined as the distance between the intermediate and superficial aponeurosis and was measured in centimetres (Cronin et al., 2021).

### Protocol

To identify the proximal and distal myotendinous junctions (MTJs), the technique described by Freitas and others was used (Freitas et al., 2018). A “mark” on the skin was used to indicate the position of the MTJs, with the distance between these two marks (in millimetres) being used to quantify muscle length. Each hamstring muscle was divided into two distinct zones; a proximal zone (zone A), and a distal zone (zone B) (Cronin et al., 2022). The proximal zone represents architecture adjacent to the proximal MTJ, whereas the distal zone represents architecture adjacent to the distal MTJ. All sonograms were acquired from the left limb only. This sonographic acquisition technique is a technically reliable (Cronin et al., 2022) and precise method of quantifying hamstring muscle architecture (Cronin et al., 2021).

### Data analysis and statistics

All statistical analysis were performed with IBM SPSS Statistics for Windows (Version 22.0, NY, United States, IBM Corp).

A Pearson product-moment correlation was used to determine the relationship between player height and fascicle length of the biceps femoris long head muscle (zone A and zone B) and the semimembranosus muscle (zone A and zone B). This was undertaken to determine whether player height should be included as a co-variate in any between-group analyses of fascicle length. Separate correlation analyses were performed for each group (female elite-level rugby union players and male elite-level rugby union players). The magnitude of correlation (r) was evaluated according to the recommendations of Hopkins (2015) as follows: r = 0.0–0.09 (trivial), r = 0.1–0.29 (small), r = 0.3–0.49 (moderate), r = 0.5–0.69 (large), r = 0.7–0.89 (very large), r = 0.9–0.99 (nearly perfect), r = 1 (perfect) (Hopkins, 2015).

Independent samples t-tests were performed to evaluate whether there were differences in fascicle length of the biceps femoris long head muscle (zone A and zone B) and the semimembranosus (zone A and zone B) muscle between female and male elite-level rugby union players. The independent variable was sex (female elite-level rugby union players vs. male elite-level rugby union players). The dependent variables were fascicle length of the biceps femoris long head muscle (zone A and zone B) and fascicle length of the semimembranosus muscle (zone A and zone B). To adjust for multiple comparisons a new *a priori* statistical significance level of *p* < 0.0125 was used. Cohen’s d effect size was calculated and interpreted in line with Cohen’s recommendations on the following descriptor scale; less than 0.20 = trivial, 0.20 = small, 0.50 = medium, 0.80 = large. 1.30 = very large (Cohen, 1992).

Independent samples t-tests were performed to evaluate whether there were differences in muscle thickness of the biceps femoris long head, biceps femoris short head, semimembranosus and semitendinosus muscles (zone A and zone B) between female and male elite-level rugby union players. The independent variable was sex (female elite-level rugby union players vs. male elite-level rugby union players). The dependent variables were muscle thickness of the biceps femoris long head, biceps femoris short head, semimembranosus and semitendinosus muscles (zone A and zone B). To adjust for multiple comparisons a new *a priori* statistical significance level of *p* < 0.006 was used. Cohen’s d effect size was calculated and interpreted in line with Cohen’s recommendations on the following descriptor scale; less than 0.20 = trivial, 0.20 = small, 0.50 = medium, 0.80 = large. 1.30 = very large (Cohen, 1992).

A Pearson product-moment correlation was used to determine the relationship between player height and pennation angle of the biceps femoris long head muscle (zone A and zone b) and the semimembranosus muscle (zone A and zone B). This was undertaken to determine whether player height should be included as a co-variate in any performed between-group analyses of pennation angle. Separate correlation analyses were performed for each group (female elite-level rugby union players and male elite-level rugby union players). The magnitude of correlation (r) was evaluated according to the recommendations of Hopkins (2015) as follows: r = 0.0–0.09 (trivial), r = 0.1–0.29 (small), r = 0.3–0.49 (moderate), r = 0.5–0.69 (large), r = 0.7–0.89 (very large), r = 0.9–0.99 (nearly perfect), r = 1 (perfect) (Hopkins, 2015).

Independent samples t-tests were performed to evaluate whether there were differences in pennation angle of the biceps femoris long head muscle (zone A and zone B) and the semimembranosus (zone A and zone B) muscle between female and male elite-level rugby union players. The independent variable was group (female elite-level rugby union players vs. male elite-level rugby union players). The dependent variables were fascicle length of the biceps femoris long head muscle (zone A and zone B) and fascicle length of the semimembranosus muscle (zone A and zone B). To adjust for multiple comparisons a new *a priori* statistical significance level of *p* < 0.0125 was used. Cohen’s d effect size was calculated and interpreted in line with Cohen’s recommendations on the following descriptor scale; less than 0.20 = trivial, 0.20 = small, 0.50 = medium, 0.80 = large. 1.30 = very large (Cohen, 1992).

Independent samples t-tests were performed to evaluate whether there were differences in (muscle) length of the biceps femoris long head muscle, the biceps femoris short head muscle, the semimembranosus muscle, and the semitendinosus muscle between female and male elite-level rugby union players. To adjust for multiple comparisons a new *a priori* statistical significance level of *p* < 0.0125 was used. Cohen’s D effect size was calculated and interpreted in line with Cohen’s recommendations on the following descriptor scale; less than 0.20 = trivial, 0.20 = small, 0.50 = medium, 0.80 = large. 1.30 = very large (Cohen, 1992).

## Results

No significant correlations were observed between player height and fascicle length of the biceps femoris long head muscle (zone A and zone B) and semimembranosus muscle (zone A and zone B) for either the female or male groups. Therefore, player height was not included as a co-variate in any analyses related to fascicle length.

No significant between-group differences in fascicle length of the biceps femoris long head muscle (zone A and zone B) and the semimembranosus muscle (zone A and zone B) were observed (Table 1).

**TABLE 1:** Fascicle length.

	Female	Male	Mean difference	95% CI of mean difference	Effect size (Cohen’s d)
Biceps femoris long head zone A	8.21 ± 0.94	8.64 ± 0.77	−0.43	−0.98 to 0.12	−0.50
Biceps femoris long head zone B	7.23 ± 1.10	7.40 ± 1.24	−0.11	−0.86 to 0.64	−0.94
Semimembranosus zone A	6.00 ± 1.10	6.10 ± 1.40	−0.07	−0.86 to 0.72	−0.56
Semimembranosus zone B	6.00 ± 1.10	5.69 ± 1.09	0.30	−0.39 to 1.00	0.28

All fascicle length values are in cm.

No significant between-group differences in thickness of the biceps femoris long head muscle (zone A and zone B), the semitendinosus muscle (zone A) and the semimembranosus muscle (zone A and zone B) were observed (Table 2). A significant difference in muscle thickness of the biceps femoris short head muscle (zone A) was observed between female and male elite-level rugby union players (mean difference = −0.66 cm; 95% CI of mean difference = −0.88 to −0.45; effect size = −1.97 (very large); *p* = < 0.001 (Table 2). A significant difference in muscle thickness of the biceps femoris short head muscle (zone B) was observed between female and male elite-level rugby union players (mean difference = −0.57 cm; 95% CI of mean difference = −0.77 to −0.36; effect size = −1.78 (very large); *p* = < 0.001) (Table 2). A significant difference in muscle thickness of the semitendinosus muscle (zone B) was observed between female and male elite-level rugby union players (mean difference = −0.39 cm; 95% CI of mean difference = −0.62 to −0.16; effect size = −1.07 (large); *p* = 0.002) (Table 2).

**TABLE 2:** Muscle thickness.

	Female	Male	Mean difference	95% CI of mean difference	Effect size (Cohen’s d)
Biceps femoris long head zone A	3.40 ± 0.34	3.67 ± 0.37	−0.27	−0.50 to −0.45	−0.77
Biceps femoris long head zone B	3.34 ± 0.33	3.55 ± 0.35	−0.21	−0.43 to 0.01	−0.62
Bicep femoris short head zone A	2.01 ± 0.31	2.68 ± 0.36	−0.66^a^	−0.88 to −0.45	−1.97
Bicep femoris short head zone B	2.05 ± 0.28	2.62 ± 0.35	−0.57^a^	−0.77 to 0.36	−1.78
Semitendinosus zone A	2.73 ± 0.43	3.02 ± 0.28	−0.29	−0.52 to −0.58	−0.80
Semitendinosus zone B	2.79 ± 0.42	3.18 ± 0.31	−0.39^a^	−0.62 to −0.16	−1.07
Semimembranosus zone A	3.56 ± 0.60	4.06 ± 0.65	−0.51	−0.91 to −0.11	−0.82
Semimembranosus zone B	3.89 ± 0.48	4.33 ± 0.53	−0.45	−0.77 to −0.13	−0.90

All muscle thickness values are in cm.

^a^ significant between group difference.

No significant correlations were observed between player height and pennation angle of the biceps femoris long head muscle (zone A and zone B) and semimembranosus muscle (zone A and zone B) for either female or male groups. Therefore, player height was not included as a co-variate in any analyses related to pennation angle.

No significant between-group differences in pennation angle of the biceps femoris long head muscle (zone A and zone B) and the semimembranosus muscle (zone A and zone B) were observed (Table 3).

**TABLE 3:** Pennation angle.

	Female	Male	Mean difference	95% CI of mean difference	Effect size (Cohen’s d)
Biceps femoris long head zone A	21.77 ± 5.39	22.00 ± 5.59	−0.23	−3.74 to 3.29	−0.04
Biceps femoris long head zone B	20.82 ± 4.22	22.37 ± 5.38	−1.55	−4.65 to 1.54	−0.32
Semimembranosus zone A	21.33 ± 4.65	24.72 ± 5.82	−3.39	−6.76 to −0.02	−0.64
Semimembranosus zone B	21.62 ± 6.04	22.40 ± 5.03	−0.77	−4.33 to 2.79	−0.14

All pennation angle values are in degrees.

A significant difference in length of the biceps femoris long head muscle was observed between female and male elite-level rugby union players (mean difference = −2.95 cm; 95% CI of mean difference = −4.03 to −1.87; effect size = −1.74 (very large); *p* = < 0 .001). A significant difference in length of the biceps femoris short head muscle was observed between female and male elite-level rugby union players (mean difference = −3.40 cm; 95% CI of mean difference = −4.98 to −1.82; effect size = −1.38 (very large); *p* = <0.001). A significant difference in length of the semitendinosus muscle was observed between female and male elite-level rugby union players (mean difference = −3.00 cm; 95% CI of mean difference = −4.73 to −1.27; effect size = −1.11 (large); *p* = 0.001). A significant difference in length of the semimembranosus muscle was observed between female and male elite-level rugby union players (mean difference = −2.80 cm; 95% CI of mean difference = −4.06 to −1.54; effect size = −1.43 (very large); *p* = < 0.001) (Table 4).

**TABLE 4:** Muscle length.

	Female	Male	Mean difference	95% CI of mean difference	Effect size (Cohen’s d)
Biceps femoris long head	29.70 ± 1.84	32.65 ± 1.53	−2.95^a^	−4.03 to −1.87	−1.74
Biceps femoris short head	23.30 ± 2.27	26.70 ± 2.64	−3.40^a^	−4.98 to −1.82	−1.38
Semimembranosus	30.25 ± 1.78	33.05 ± 2.14	−2.80^a^	−4.06 to −1.54	−1.43
Semitendinosus	33.60 ± 3.15	36.60 ± 2.16	−3.00^a^	−4.73 to −1.27	−1.11

All muscle length values are in cm.

^a^ significant between group difference.

## Discussion

This study investigated whether differences exist in the architectural characteristics of the hamstring muscles of elite-level male and female rugby players. Our analyses revealed that there were no significant between-group differences in fascicle length and pennation angle. We did observe significant between-group differences in muscle thickness of the semimembranosus muscle (zone B) and bicep femoris short head muscle (zone A and B); the male players had thicker muscles. Additionally, the elite-level male players had longer hamstring muscles - for all muscles evaluated.

With respect to fascicle length, our observations are similar to those of Behan et al. (2019), who reported that the fascicle length of the biceps femoris long head muscle does not differ between recreationally active men and women. Fascicle length and changes in fascicle length of the bicep femoris long head muscle have been reported to associate with HSI and risk of reinjury in male athletes (Timmins et al., 2016a; Timmins et al., 2016b). One retrospective study observed fascicle length to be 1.54 cm shorter in the injured bicep femoris long head muscle (10.40 cm) when compared to the uninjured bicep femoris long head muscle (11.94 cm) (Timmins et al., 2015). Indeed, a prospective study identified that athletes who possessed “short” bicep femoris long head muscle fascicle lengths (<10.54 cm) were 4.1 times more likely to sustain future HSI, whereby for every 0.5 cm increase in bicep femoris long head muscle fascicle length, the risk of HSI was reduced by 73.9% (Timmins et al., 2016a). Our data indicate that at a group-level, fascicle length of the biceps femoris long head muscle and semimembranosus muscle do not differ between male and female elite-level rugby union players. Therefore, at a group-level, fascicle length is unlikely to be a sex-specific intrinsic risk factor for HSI.

We observed significant between-group differences in muscle thickness of the bicep femoris short head muscle (zone A and zone B). The between-group differences were 0.66 cm (zone A) and 0.57 cm (zone B), respectively; the male athletes had thicker muscles. In a previous study we described that the standard error of measurement (SEM) associated with the ultrasound technique for the quantification of muscle thickness of the bicep femoris short head muscle was 0.08 cm for zone A and zone B (Cronin et al., 2022). Therefore, we can be confident that our observed significant between-group difference in muscle thickness of the bicep femoris short head muscle (zone A and zone B) represents a true difference (as it exceed the SEM). We also observed a significant between-group differences in muscle thickness of the semitendinosus muscle (zone B). The between-group difference was 0.39 cm. In a previous study we described that the standard error of measurement (SEM) associated with the US technique for the quantification of muscle thickness of the semitendinosus muscle was 0.09 cm for zone B (Cronin et al., 2022). Therefore, we can be confident that our observed significant between-group difference in muscle thickness of the semitendinosus muscle (zone B) represents a true difference (as it exceed the SEM). The prevalence of biceps femoris short head muscle injuries reported in the published literature is low. In a retrospective study with 275 male soccer players who had sustained HSI; the bicep femoris long head muscle was the most commonly injured (56.5%), followed by the semitendinosus muscle (24.4%), semimembranosus muscle (13.7%), and bicep femoris short head muscle (5.6%) (Crema et al., 2015). In elite-level male rugby union players, a recent study highlighted that only 12% of injuries involve either the biceps femoris short head muscle or semitendinosus muscle (Kerin et al., 2022). To our knowledge, no published literature has reported upon the physiological association between muscle thickness and the risk of HSI. Although we observed significant between-group differences in muscle thickness of the bicep femoris short head muscle (zone A and zone B) and semitendinosus (zone B) muscle, we acknowledge that the differences, although likely to be true differences, are somewhat small (0.30–0.49 cm). It is important to understand that ultrasound is highly operator dependent when assessing skeletal muscle architecture (Carr et al., 2021), therefore any minor changes to transducer orientation (Ishida et al., 2018; Dankel et al., 2020) and pressure applied to the skin (Treece et al., 2002) will influence muscle thickness measurements.

We observed significant differences in the length of the hamstring muscles between male and female elite-level rugby union players (Table 4). This was somewhat expected, as male athletes were significantly taller (*p* = <0.001); the mean height of the male players was 1.9 m while the mean height of the female players was 1.7 m. However, no significant correlations were observed between player height and fascicle length of the biceps femoris long head muscle (zone A and zone B) and semimembranosus muscle (zone A and zone B) for either groups. Indeed, we observed no significant correlations between player height and pennation angle of the biceps femoris long head muscle (zone A and zone B) and semimembranosus muscle (zone A and zone B) for either groups. Lieber and others identified that longer fascicle lengths contribute to an increase in muscle length and muscle velocity, whereas shorter fascicles limit muscle length and muscle velocity (Lieber, 1993). The majority of studies assessing the associations between hamstring muscle architectural characteristics and injury/re-injury have focussed on the geometric distribution of the fascicles and fascicle length changes (Timmins et al., 2016a; Timmins et al., 2017; Whiteley et al., 2022). However, to our knowledge an association between hamstring muscle length and the risk of HSI has not been described in the published literature. Furthermore, the there is no direct association between hamstring muscle length and HSI and our findings of significant increases in hamstring muscle lengths between male and female elite level rugby union players does not at least independently explain the higher incidence of HSI in males when compared to females.

We acknowledge that our cross-sectional study design is a limitation; we only assessed the architectural characteristics of the hamstring muscles at a single point in time. Future studies should include prospective designs which track the natural fluctuation of the hamstring muscle architectural characteristics over the course of a full competitive season. We acknowledge the lower sample size of our study. Future studies should be undertaken on full team player cohorts and not a convenience sample of players.

## Conclusion

Our study is the first to comprehensively compare and contrast the architectural characteristics of the hamstring muscles of elite-level male and female rugby union players. At a group-level, no between-group difference in fascicle length or pennation angle were observed. Thus, at a group level, hamstring muscle fascicle length and pennation angle are unlikely to be a sex-specific intrinsic risk factor for HSI.

## Data Availability

The datasets presented in this article are not readily available because of an authorial declaration to the Research Ethics Committee at University College Dublin, Ireland, that precludes the authors from making the raw data publicly available. Requests to access the datasets should be directed to kevin.cronin@ucd.ie.
